# Polygenic risk scores for autoimmune related diseases are significantly different in cancer exceptional responders

**DOI:** 10.1038/s41698-024-00613-x

**Published:** 2024-05-25

**Authors:** Siyuan Chen, Amelia L. M. Tan, Maria C. Saad Menezes, Jenny F. Mao, Cassandra L. Perry, Margaret E. Vella, Vinayak V. Viswanadham, Shilpa Kobren, Susanne Churchill, Isaac S. Kohane

**Affiliations:** 1 Department of Biomedical Informatics, Harvard Medical School, 10 Shattuck Street, Boston, MA 02115 USA; 2 Department of Biostatistics, Harvard T.H. Chan School of Public Health, 677 Huntington Avenue, Boston, MA 02115 USA; 3 Department of Computer Science, Yale University, 51 Prospect Street, New Haven, CT 06511-8937 USA

**Keywords:** Cancer genomics, Cancer therapy

## Abstract

A small number of cancer patients respond exceptionally well to therapies and survive significantly longer than patients with similar diagnoses. Profiling the germline genetic backgrounds of exceptional responder (ER) patients, with extreme survival times, can yield insights into the germline polymorphisms that influence response to therapy. As ERs showed a high incidence in autoimmune diseases, we hypothesized the differences in autoimmune disease risk could reflect the immune background of ERs and contribute to better cancer treatment responses. We analyzed the germline variants of 51 ERs using polygenic risk score (PRS) analysis. Compared to typical cancer patients, the ERs had significantly elevated PRSs for several autoimmune-related diseases: type 1 diabetes, hypothyroidism, and psoriasis. This indicates that an increased genetic predisposition towards these autoimmune diseases is more prevalent among the ERs. In contrast, ERs had significantly lower PRSs for developing inflammatory bowel disease. The left-skew of type 1 diabetes score was significant for exceptional responders. Variants on genes involved in the T1D PRS model associated with cancer drug response are more likely to co-occur with other variants among ERs. In conclusion, ERs exhibited different risks for autoimmune diseases compared to typical cancer patients, which suggests that changes in a patient’s immune set point or immune surveillance specificity could be a potential mechanistic link to their exceptional response. These findings expand upon previous research on immune checkpoint inhibitor-treated patients to include those who received chemotherapy or radiotherapy.

## Introduction

In cancer trials, there is a small number of patients who have meaningful and long-lasting responses to treatment that greatly exceed historical norms. Historically, these rare patients have been declared as unexpected outliers, and the molecular basis behind their therapeutic successes has not been thoroughly investigated. However, the advent of whole genome sequencing (WGS) has increased interest in mechanisms responsible for these remarkable outcomes.

Important insights have emerged regarding the mechanisms that may explain the exceptional response of some cancer patients. Some of these mechanisms include genetic variation and unique responses to DNA damage pathways, crucial mutations in cancer driver genes or tumor suppressor genes, diversity in intracellular signaling, variations in immune engagement and special gene fusion events^1–5^. For example, a patient with metastatic urothelial cancer who responded exceptionally well to nivolumab after unsuccessful attempts with chemotherapy, radiation, and surgery, was found to have elevated mRNA levels of the genes responsible for encoding PD-1 and PD-L1. In addition, there was a 32x amplification in IFNG, leading to extremely high expression of the gene in both cancer cells and T cells, which were considered potential explanations for the exceptional response by Wheeler et al^1^. This demonstrates how alterations in the tumor’s immune microenvironment can account for a patient’s favorable response to a specific treatment. In addition, germline genetic factors are important modifiers in tumor immune microenvironment, cancer risk and immunotherapy response, which can be investigated by polygenic risk scores^6,7^.

In 2018, to further learn from these exceptional cancer survivors, we launched the registry for the Network for Enigmatic Exceptional Responders (NEER) across all trials as a patient-directed study so that any patient could volunteer their own case and data as a candidate for the NEER registry. The first 53 patients who survived 2 standard deviations greater than the survival rate or exhibited significant deviation from standard clinical treatment (see methods) were enrolled and sequenced. The registry contains a broad collection of cancer types (Table 1) and a range of treatments including chemotherapy, radiation therapy and immunotherapy (Supplementary Table 1). Here, we describe the results of our first study examining the germline genomes of NEER participants in search of shared genetic tendencies towards cancer exceptional responses. As the contribution to an exceptional response of any given common variant is going to be small, we used previously published polygenic risk scores (PRS) to compare the genetic risk for autoimmune diseases between exceptional responders (ERs) and typical cancer patients^8–12^. Prior studies have reported that elevated PRSs for autoimmune diseases such as hypothyroidism in patients receiving immunotherapy are associated with autoimmune adverse events and are variably associated with improved outcomes^8,13^. Additionally, genetic variants associated with autoimmune diseases based on GWAS analysis were reported to alter cancer immunity and IFN signaling^14^. Interestingly, in our cohort of ERs, 20% of patients have been diagnosed with autoimmune diseases and some of them also have first-degree relatives with autoimmune diseases. Therefore, we hypothesize the inherited germline genetics of ERs related to autoimmune diseases could have an impact on a favorable therapeutic response. The difference in PRSs of autoimmune diseases may reflect how common variants contribute to the general immune functions of individuals and affect cancer prognosis. Rather than generating a new set of scores based on a single reference population, we decided to use previously published PRSs to directly compare findings in NEER with the claims of the prior studies. These previous reports hypothesized that autoimmunity led to better outcomes for cancer treatment by modulating the overall reactivity of helper T cells and B cells. To build upon this hypothesis and investigate which autoimmune processes could offer a potential explanation for favorable therapeutic response, we conducted PRS analysis for 7 different autoimmune-related diseases (Supplementary Table 2). Additionally, while the previous studies largely focused on patients with immunotherapy treatments, we decided to include the full range of treatments in NEER, allowing us to determine whether the improvement was present in patients treated with chemotherapies and other non-immunomodulating therapies.

**Table 1:** Demographic summary of NEER and PCAWG subset

	NEER	PCAWG
*N*	51	414
Male, n(%)	20 (39.2%)	154 (37.2%)
Age, median (IQR)	66 [62,70]	62 [51,71]
*Organ System, n(%)*		
Breast	13 (25.5%)	106 (25.6%)
Pancreas	7 (13.7%)	57 (13.8%)
Lung	5 (9.8%)	41 (9.9%)
Leukemia	4 (7.8%)	33 (8.0%)
Ovary	4 (7.8%)	33 (8.0%)
Kidney	3 (5.9%)	24 (5.8%)
Uterus	3 (5.9%)	24 (5.8%)
Brain	2 (3.9%)	16 (3.9%)
Intestine	2 (3.9%)	16 (3.9%)
Melanoma	2 (3.9%)	16 (3.9%)
Bladder	1 (2.0%)	8 (1.9%)
Bone	1 (2.0%)	8 (1.9%)
Esophagus or Stomach	1 (2.0%)	8 (1.9%)
Gallbladder or Liver	1 (2.0%)	8 (1.9%)
Prostate	1 (2.0%)	8 (1.9%)
Thyroid	1 (2.0%)	8 (1.9%)
*Cancer Stage, n(%)*		
Binet A	0 (0.0%)	18 (4.3%)
Binet B	0 (0.0%)	4 (1.0%)
Binet C	1 (2.0%)	2 (0.5%)
I	0 (0.0%)	42 (10.1%)
II	0 (0.0%)	59 (14.3%)
III	2 (3.9%)	31 (7.5%)
IV	43 (84.3%)	3 (0.7%)
NA	5 (9.8%)	225 (61.6%)
*Vital Status, n(%)*		
Alive	47 (92.2%)	266 (64.3%)
Deceased	4 (7.8%)	126 (30.4%)
NA		22 (5.3%)
Years survived (censored), median (IQR)	12.77 [9.01,18.04]	2.89 [1.35,5.21]

## Results

### Characteristics of the NEER exceptional responders

The two outliers of NEER ERs in terms of genetic ancestry were excluded in the following analysis and a comparable control set was identified according to cancer site and ancestry population (see methods) based on the clinical information and germline WGS data collected by the Pancancer Analysis of Whole Genomes (PCAWG) project^15^. PCAWG was selected as it comprises a large heterogeneous set pan-cancer cohort with WGS. The NEER participants were 51 cancer ERs with different cancer types. Of these 51 participants, a majority of them were breast cancer patients (26%), followed by pancreatic cancer patients (14%), and lung cancer patients (10%). Notably, a high proportion of the ERs were diagnosed with distant metastasis and stage IV disease, accounting for 84% of the total. There are four ERs of whom staging was not applicable (one with primary plasma cell leukemia, one with acute myeloid leukemia, and two with glioblastomas) because the TNM or Binet staging systems are not typically used for these types of cancer. Despite the advanced disease status of the NEER cohort, the median survival duration is a remarkable 12.77 years, highlighting their exceptional survival trajectories (Table 1). The detailed demographic, clinical and drug assignment data of 51 ERs can be found in Supplementary Table 3.

ERs underwent a variety of therapies, including surgery, chemotherapy, hormone therapy, immunotherapy, radiation and targeted therapy. The vast majority (78%) underwent at least one surgical procedure focused on the primary tumor or distant metastasis. A heterogeneous set of chemotherapies protocols were administered to 75% of the patients. Radiation therapy was another common treatment modality, with 59% of patients receiving it. Nearly half of the patients (45%) were also treated with targeted therapy. Hormonal therapy and immunotherapy were less frequently administered at 22% and 14% respectively (Supplementary Table 1).

In the NEER group, 10 participants (20%) were confirmed to have autoimmune diseases (Supplementary Table 3), while the rate of having at least one autoimmune disease is about 4% worldwide and in the US is around 8%^16^. Of these 10 patients, eight had a single autoimmune diagnosis: Crohn’s disease (3), multiple sclerosis (MS) (3), Graves’ disease (1), and antiphospholipid syndrome (1). The remaining two patients had dual diagnoses: one with Hashimoto’s disease and lichen planus, and the other with autoimmune hepatitis and nephritis. Concerning familial predisposition, 16% of the NEER participants had a first-degree relative with an autoimmune disease. The autoimmune disease rate of PCAWG typical cancer patients is not available.

### Differences in PRSs between NEER and PCAWG

**Fig. 1:**
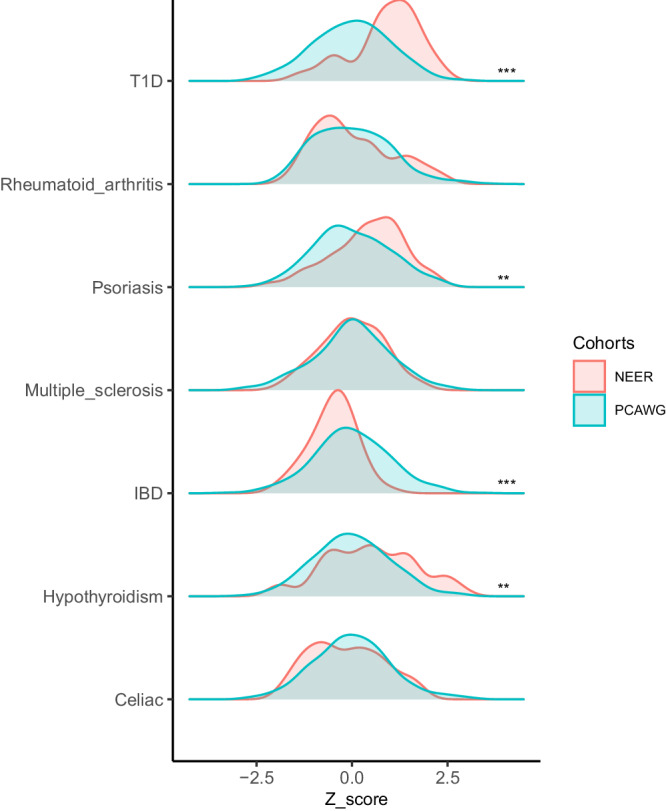
Distribution of PRS Scores in NEER and PCAWG across 7 autoimmune/inflammatory-related disorders. The asterisks denote significance per the Wald test. * signifies *p* < 0.05 **p < 0.01 ***p < 0.001.

We implemented several previously published PRS models corresponding to seven autoimmune disorders, including type 1 diabetes (T1D)^9^, rheumatoid arthritis (RA)^10^, psoriasis^10^, MS^11^, inflammatory bowel disease (IBD)^12^, hypothyroidism^8^, and celiac disease^10^. The ERs showed significantly higher PRSs for hypothyroidism (OR = 1.58, CI = [1.19, 2.11], *p* = 0.002), T1D (OR = 2.66, CI = [1.90, 3.81], *p* < 0.001) and psoriasis (OR = 1.52, CI = [1.13, 2.06], *p* = 0.006) risk. In contrast, there were significantly lower PRSs for IBD (OR = 0.58, CI = [0.42, 0.79], *p* < 0.001) risk compared to typical cancer patients (Fig. 1, Supplementary Fig. 1). RA, MS and celiac PRSs were not significantly different.

In order to know whether the PRSs generalized to prognosis in a wide range of typical cancer patients, for each of the PRSs we also checked the survival curves of all PCAWG patients when stratified by high vs low PRS (see Methods). These are illustrated in Supplementary Fig. 2. None of the differences were statistically significant, but those cancer patients with high hypothyroidism risk tend to live longer (*p* = 0.078), which is consistent with our analysis in ERs.

The PRS should approximate a normal distribution when the sample size is adequate, but the skewness of the PRS distribution may also reflect some special characteristics of a small group of people. All PRSs in the large cohorts, the typical cancer patients, are not skewed except the one for RA, which had a significant right-skew (Table 2). With a much smaller sample size in NEER, the NEER T1D PRS distribution was left-skewed (Table 2), which means the distribution of the scores is asymmetrical with enrichment for scores that are on the higher end and a larger proportion of ERs had significantly higher T1D PRSs. As the distribution of the PCAWG T1D PRS scores is not skewed, this indicates that many NEER patients were at higher genetic risk of T1D than typical cancer patients in PCAWG. There are 42 ERs having higher T1D PRSs than the mean of T1D PRS of typical cancer patients.

**Table 2:** Skew of distribution from normal in PRS of each population

PRS Score Skew p value	NEER	PCAWG
Hypothyroidism	0.977	0.299
T1D	0.02	0.892
Multiple_sclerosis	0.788	0.21
Psoriasis	0.134	0.28
IBD	0.472	0.683
Rheumatoid_arthritis	0.079	0.001
Celiac	0.513	0.099

### Differences in PRSs observed with stratification

**Fig. 2:**
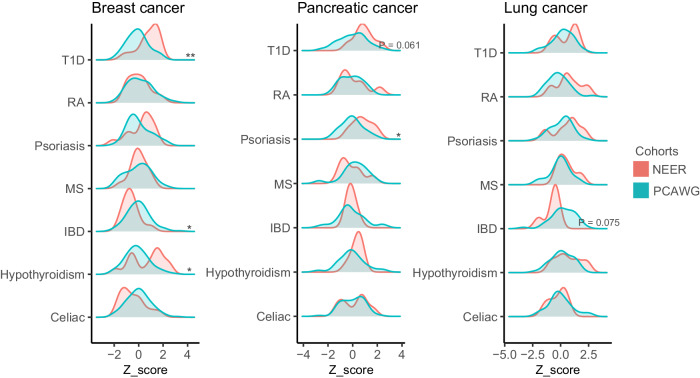
PRS distributions in patients with breast cancer, pancreatic cancer and lung cancer. The asterisks denote significance per the Wald test. * signifies *p* < 0.05 ** *p* < 0.01 *** *p* < 0.001.

Differences in PRSs for autoimmune diseases were observed when stratifying ERs by cancer type or treatment, although sample sizes were small (Fig. 2). Breast cancer ERs had higher PRSs for T1D (OR = 3.12 CI = [1.57, 6.85] *p* = 0.002) and hypothyroidism (OR = 1.96, CI = [1.15, 3.48], *p* = 0.015), but lower PRS for IBD (OR = 0.44, CI = [0.20, 0.88], *p* = 0.028) compared to typical breast cancer patients. Pancreatic cancer ERs showed higher PRSs for T1D (OR = 2.21, CI = [1.03, 5.57], *p* = 0.061) and psoriasis (OR = 2.90, CI = [1.16, 8.50] *p* = 0.030). Lung cancer ERs had a lower PRS for IBD (OR = 0.45, CI = [0.17, 1.04], *p* = 0.075). When stratifying by treatment type, ERs receiving radiation and chemotherapy consistently had higher hypothyroidism PRSs, suggesting the autoimmune-cancer drug response link extends beyond immune checkpoint inhibitors.

**Fig. 3:**
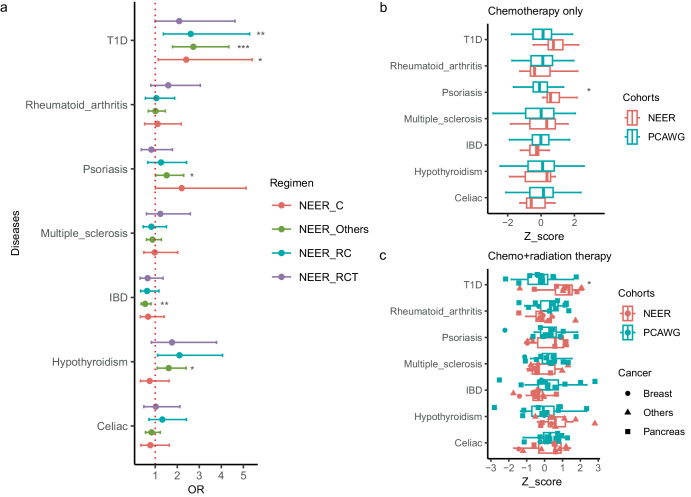
PRS distributions stratified by regimens. **a** Odds ratios (abbreviated as OR) and confidence intervals were plotted, compared to 414 PCAWG typical cancer patients. Different regimens, combinations of therapies, were labeled by different colors. NEER_C (*n* = 7), NEER_RC (*n* = 10) and NEER_RCT (*n* = 7) refer to chemotherapy only, a combination of radiation therapy and chemotherapy, and a combination of radiation therapy, chemotherapy and targeted therapy respectively among NEER ERs. NEER_Others refers to the group of ERs with other combinations of therapies. Each specific combination has a sample size less than 7. **b** ERs in NEER (*n* = 7) and patients in PCAWG (*n* = 52) treated only by chemotherapy were subsetted. Boxplots showed the distributions of their PRSs in different autoimmune diseases. **c** ERs in NEER (*n* = 10) and patients in PCAWG (*n* = 11) treated by both chemotherapy and radiation therapy were subsetted. Boxplots showed the distributions of their PRSs in different autoimmune diseases. The histological cancer types were labeled through the shapes of dots. * signifies *p* < 0.05 **p < 0.01 ***p < 0.001.

ERs were also stratified by whether they received a specific type of therapy, with similar differences observed (Supplementary Fig. 3a–e). Given that many ERs have multiple treatments, we also stratified ERs with their regimen, the combinations of treatment types. NEER ERs with different regimens consistently showed increased PRSs for T1D and decreased PRSs for IBD. ERs receiving radiation and chemotherapy consistently had higher hypothyroidism PRSs, suggesting the autoimmune-cancer drug response link extends beyond immune checkpoint inhibitors. (Fig. 3a, Supplementary Fig. 3f).

Among chemotherapy-only treated ERs (*n* = 7) versus PCAWG typical cancer patients (*n* = 52), ERs had significantly higher psoriasis PRS (OR = 4.33, CI = [1.61,18.09], *p* = 0.014) (Fig. 3b). T1D PRS was elevated (OR = 2.19, CI = [0.96, 5.62], *p* = 0.074) but not statistically significant, likely due to limited sample size. For the chemotherapy and radiation group (10 ERs, 11 PCAWG), ERs had significantly higher T1D PRSs (OR = 3.17, CI = [1.30, 10.80], *p* = 0.027) (Fig. 3c). Hypothyroidism PRS was increased and IBD PRS decreased, but not significantly. Notably, most PCAWG patients in this group had pancreatic cancer. Examining just the pancreatic cancer ERs (*n* = 3) and one breast cancer ER revealed their T1D PRSs were higher than the upper PCAWG quantile (Fig. 3c).

In summary, despite small sample sizes, ERs stratified by cancer type and treatment consistently showed altered autoimmune disease PRSs, especially the elevated T1D PRS, suggesting a link between genetic autoimmune risk and exceptional drug response.

### Genes involved in the T1D PRS model related to cancer drug response may influence exceptional responses

Because the T1D PRS model only includes 66 SNPs and shows the most significant difference between NEER and PCAWG, we investigated the SNPs and eQTLs to find whether they are associated with high T1D PRS in ERs. The allele frequencies of every single variant in the model were compared between NEER and PCAWG. There are 5 variants showing different frequencies between NEER and PCAWG, with 4 of them implicated in risk of T1D and were enriched in NEER, but none of them are of significance after multiple testing corrections. Thus, it is the accumulative effect of multiple common variants in the model that may influence the immune background of ERs. The genes involved are *TUFM*, *C11orf21*, *OAS1* and *FUT2* (Supplementary Table 4).

**Fig. 4:**
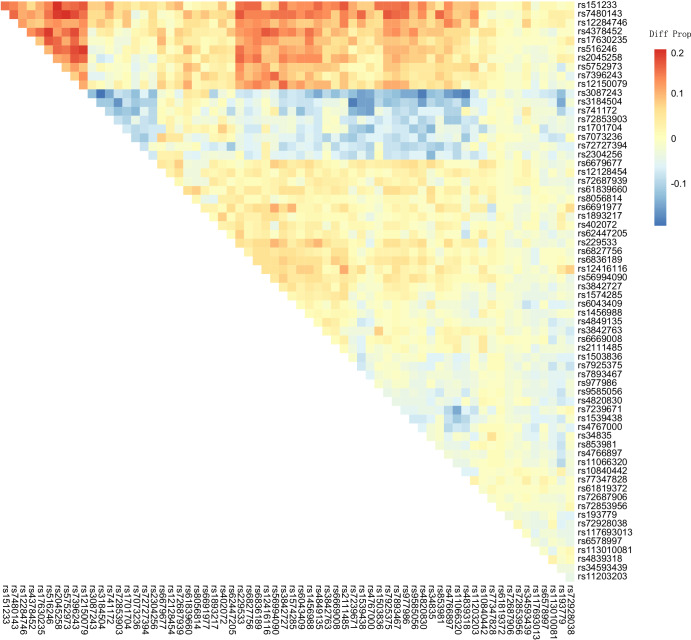
Difference of variant co-occurrence in the T1D PRS model between NEER and PCAWG. The values represent the co-occurrence score per variant pair in NEER minus the one in PCAWG, where the co-occurrence score is the proportion of patients carrying both variants in a given cohort. If values are positive, the linkage is stronger in ERs. The data is not scaled when drawing the heatmap and is clustered by both rows and columns.

Besides the effects of the single variants, we are interested in the differences in co-occurrence patterns between NEER and PCAWG. The heatmap shows the difference in the proportion of patients carrying the two variants in the T1D PRS model between NEER and PCAWG, with the cells in red indicating greater linkage of variants in NEER ERs. Variant rs3087243, a protective variant of *CTLA4*, was more likely to occur simultaneously with other variants in PCAWG, with differences greater than 10% in 22 variants (Fig. 4). As *CTLA4* has been specifically implicated in cancer immune and immune checkpoint inhibitor responses^17^, such differences in co-occurrence may influence different immune responses in exceptional responders. In addition, co-occurrence between the variant on *CENPW* (rs2045258), identified as an indicator for cancer development and prognosis in different cancer types^18,19^, and other variants in NEER were observed (Fig. 4). Our findings in variant linkage begin to characterize the cumulative effect of inherited genetic differences between typical cancer patients and ERs.

## Discussion

We collected germline WGS data from 53 ERs with different cancer types and treatments and investigated the effect of common germline variants in ERs by calculating their PRSs for key autoimmune diseases. Two NEER patients were removed from the analysis because their common variant profiles diverged from those of European origin, which most published PRS profiles have been computed, although this is likely to change soon^20,21^. A subset of patients from PCAWG, with matched population ancestry, sex, and cancer types, was used as control.

Compared to typical cancer patients in the PCAWG dataset, the cohort of NEER patients had significantly increased PRSs for T1D, hypothyroidism, and psoriasis. The ERs were also found to have significantly lower IBD PRSs. Similar results were observed when we stratified cancer patients by breast cancer, the largest group of ERs with a specific cancer type. In addition, stratifications were performed on both single therapy and combinations of therapies. Differences could be observed in risks of T1D, hypothyroidism, IBD, and psoriasis, though the sample sizes are limited.

Divergence in risk profiles for the same common variants between IBD and T1D has been noted in a study by Wang^22^ at multiple loci. For example, protein tyrosine phosphatase non-receptor type 22 (*PTPN22*), a gene that negatively regulates T cell activation, is associated with many autoimmune diseases but in opposite directions. In those studies, divergence was with respect to autoimmune disease, not cancer outcome. Whether the divergence arises from a different immune set point or different (micro)environmental stimuli remains unknown. In addition, it was reported that the autoimmune susceptibility variant on PTPN22, which inhibits phosphatase activity, could improve cancer outcomes^23^.

A previous paper showed a link between germline higher risk of T1D and lower risk of IBD and tumor interferon response^14^. Interestingly, 36 ERs have a T1D PRS greater than the mean of typical cancer patients but with low IBD PRS at the same time. Several SNPs on *PTPN2* and *IL21*, two genes involved in interferon signaling pathways, were included in the T1D PRS model. Our findings suggest that genes associated with the risk of autoimmune diseases, especially those involved in interferon response, might explain in part cancer drug response and can be used as potential prognostic markers.

The T1D PRS distribution of ERs is left-skewed. Understanding which SNPs drive the high T1D risk in part of ERs can help to understand their immune background. Thus, 51 ERs were stratified by T1D PRS > 0 (*n* = 42) or T1D PRS < 0 (*n* = 9), where 0 indicates the mean of T1D PRS in typical cancer patients, and the allele frequencies were compared. Two protective and two susceptible variants associated with T1D are distributed differently between T1D PRS high and T1D PRS low groups (Supplementary Table 5). These variants are located in genes reported to be associated with cancer drug responses suggesting a potential mechanism for heterogeneity among ERs. For instance, *MAGI3* degraded c-Myc and acts as a predictor for chemotherapy response in colorectal cancer^24^ and BCL2L15/Bfk pro-apoptotic factor was found selectively expressed in the 5-Fluorouracil responder CRC cells^25^, which was one of the chemotherapy treatments for ERs.

Prior studies of the impact of elevated or decreased PRS scores have focused on checkpoint inhibitors, specifically PD-1/PD-L1 blockage. The results have consistently shown association with new-onset autoimmune adverse events (e.g. hypothyroidism, colitis, hypophysitis) but have been less consistent in relating these to outcomes. A study of non-small cell lung cancer by Luo et al.^13^ did not reveal that a high PRS for hypothyroidism resulted in a benefit to survival. In contrast, in a study by Khan et al.^8^ there was a survival benefit for patients with triple-negative breast cancer. Another study by Khan et al.^26^ showed long overall survival for patients with bladder cancer high PRS for psoriasis and low PRS for atopic dermatitis.

Unlike the aforementioned studies, in NEER ERs, there are only 4 patients who received checkpoint inhibitors, but all of them were treated with other kinds of cancer therapies as well. Moreover, excluding those 4 patients does not change the PRS distribution of ERs in the comparison. Yet, over the NEER patients’ wide range of treatments and cancers, there were still significant differences in the distribution of PRSs for hypothyroidism and psoriasis. The ERs had significantly higher T1D PRSs and significantly lower scores for IBD than typical PCAWG cancer patients. Survival analysis for high and low PRS scores in these same autoimmune/inflammatory diseases did not reveal any significant difference in survival times.

By design, this study focused on testing a hypothesis about germline differences in common variants for autoimmune disease risk. Therefore, it cannot account for differences in the somatic genome of the tumor, or rare variants which individually might have a greater effect on treatment outcomes. It also does not address differences in lifestyle and socioeconomic status which is documented for NEER patients but not the large comparison populations like PCAWG. The study has limitations, including a small sample size and a retrospective design comparing the NEER registry to the PCAWG dataset, which may introduce biases and potential confounders, as well as the potential for survival bias in the comparison between the two datasets. As the PCAWG dataset lacks clear and comprehensive regimen data, unlike the NEER dataset, it is difficult to match controls perfectly based on both tumor type and treatment regimen to minimize confounding from varying survival rates. Nonetheless, our work demonstrates the reproducibility of the PRS findings from earlier studies and presents significant PRS findings for additional autoimmune diseases such as T1D in a dataset containing various tumor types and cancer therapies other than checkpoint inhibitors. These findings support the hypothesis of a mechanistic link between more prevalent germline variants in NEER patients and their exceptional response to cancer treatment.

## Methods

### Cancer exceptional responders sample collection

NEER ERs were obtained from a group of US-based applicants aged at least 18 years old with an exceptional response to cancer therapies. Of 222 individuals who registered for the study, 82 were accepted based on their eligibility. Eligibility was generally determined based on the most recently available survival means within each cancer type, with eligible participants exceeding 2 standard deviations greater than the survival rate or exhibiting significant deviation from standard clinical treatment. There was a combination of sources that we used to determine the current cancer survival rates. The ones most frequently used were those available through the American Cancer Society^27^. When these were not applicable, recent publications for listed survival rates or other well-known sources were used^28^. Some of the participants were further evaluated by cancer-specific oncology experts (see Acknowledgements) to ensure that the ER candidates were indeed outliers, based on their presentation and course, before they were enrolled. The study inclusion reasons of those who were evaluated individually can be found in the supplementary methods. Electronic health records and blood samples were collected from 53 ERs at the time of this analysis. The Institutional Review Board (IRB) of Harvard Faculty of Medicine gave ethical approval for this work. Individuals consented to participation using a written consent form that was signed digitally. All relevant ethical regulations including the Declaration of Helsinki have been compiled. All participants provided informed consent for themselves – none were deceased at the time of enrollment. The study was publicly posted on the people-powered medicine website (https://peoplepoweredmedicine.org/neer). Whole blood samples of participants were used for whole genome sequencing (WGS) with 30X mean coverage at the Broad Institute. DNA was extracted from aliquots of whole blood using the QIAsymphony DSP DNA Kit in conjunction with the QIAsymphony SP instrument (Qiagen). DNA was processed for PCR-Free library construction, sequenced with 150 bp paired-end reads, and sample identification QC check. A KAPA HyperPrep library preparation was followed by qPCR quantification.

### Data processing

WGS data was processed through the CGAP pipeline developed by Harvard’s Department of Biomedical Informatics and Brigham Genomic Medicine. Variants were filtered through GQ > 20 and VQSR as quality controls. The NEER dataset assembly is based on the GRCh38 genome but most PRS models were from GRCh37. A liftover procedure^29^ was used to convert datasets from one genome assembly to another to make them comparable. Minimac4 1.5.7 genotype imputation^30^ was applied, based on Haplotype Reference Consortium (HRC) panel, to increase power and improve the PRS^31,32^. Variants from sex chromosomes were excluded and among imputed SNPs, only those with *R*^2^ > 0.8 were retained. Furthermore, we set the threshold for the P-value of the Hardy–Weinberg test at 0.001 and variants genotyped in <90% of the samples were removed. An ancestry population check was performed through PCA based on all shared variants between NEER ERs and PCAWG typical cancer patients after NEER data went through the genomic liftover procedure and was imputed.

Two NEER participants (52 and 53) were found to be distant from a cluster of the other 51 participants in principal component analyses based on SNPs (Supplementary Fig. 4). They also carried the largest number of variants and the largest number of homozygous variants and were identified as having a non-European continent of origin. Earlier PRS models have been shown to be brittle in their performance when applied to populations differing from those they were developed on^10,12,33–35^ even though recent methods and data sources are resulting in greater robustness^20,21^. As most of the PRS models were developed on European populations, only the 51 NEER ERs were included.

The germline jointly genotyped WGS dataset of PCAWG went through the same quality control filter and only the population of European origin was retained. After excluding PCAWG participants of non-European genomic ancestry, the PCAWG dataset was matched with the NEER dataset to achieve equivalent diversity and proportion of cancer organ sites. To accomplish this, the PCAWG dataset was randomly downsampled until the proportions aligned (Table 1).

### Polygenic risk score calculation

PRS is calculated as a sum of weighted effect alleles. The general mathematical formula of the PRS is written as follows:

1 $${PRS}=\mathop{\sum }\limits_{i=1}^{n}{w}_{i}{\rm{\cdot }}{X}_{i}$$

where X_i_ denotes the effect allele count and w_i_ denotes the weight of the *i*th SNP for a specified outcome. The number of SNPs included in PRS varies, depending on the trait/disease, and was determined in the earlier studies^8,9,11^ (see Supplementary Table 2). When comparing PRSs between NEER and PCAWG, only variants shared between the two cohorts were included. We collected autoimmune diseases with publicly available PRS models that were reported to be associated with cancer risk or cancer drug response. The model should be built or developed mostly by the European population. Thirdly, most of the SNPs involved in the models should be detected in both NEER and PCAWG data. The PRS models implemented and the number of SNPs in each model are listed in the Supplementary Table 2.

### Survival analysis

A Kaplan–Meier (KM) analysis was performed among all PCAWG patients. Patients with missing data on vital status were excluded. Patients were divided into high-risk groups and low-risk groups based on the 10% and 90% quantile of PRSs. A log-rank test was used to compare the difference in survival time in the KM curve.

### Logistic regression

Wald tests of logistic regression coefficients were used as statistical tests between two groups of PRSs. The logistic regression model does not include covariates to adjust the confounding effects as we have confirmed that all the samples analyzed were of the same ancestry group and similar demographic background. The results were displayed by odds ratio per unit of normalized PRS, confidence intervals, and p-values from the logistic regression. All tests were two-tailed tests at α < 0.05 level. All statistical analysis was performed using R 3.6.1.

### Skew analysis

While the difference in distribution test above compares PCAWG to NEER, it does not test whether the PRS scores are skewed within each population individually. The estimate of skew is based on the sample skewness^36^ b_1_ where

2 $${b}_{1}=\frac{\,\frac{1}{n}{\sum }_{i=1}^{n}{\left({x}_{i}-{\underline{x}}\right)}^{3}}{{\left[\frac{1}{n-1}{\sum }_{i=1}^{n}{\left({x}_{i}-{\underline{x}}\right)}^{2}\right]}^{\frac{3}{2}}}$$

This estimate was calculated for PCAWG and NEER using the skewness.norm.test function in the normtest package in R.

### Analysis on SNPs in T1D PRS model

The allele frequency of the given variant was first calculated based on the sample size. Then, Fisher’s exact tests were applied to every single variant to compare the different allele frequencies between different groups of individuals. Benjamini-Hochberg method was used for multiple testing corrections. When analyzing the co-occurrence of variants, how many patients who carried the given two variants were counted in both cohorts. Differences in the proportion between NEER and PCAWG were then visualized in the heatmap.

### Supplementary information

Supplementary Information

## Data Availability

Because submitting the data to a public repository was not allowed through the original IRB protocol, the SNPs data that support the findings of this study are available in DBMI Data Portal (https://portal.dbmi.hms.harvard.edu/projects/ppm-neer) upon reasonable request. The PCAWG data can be obtained through the ICGC Data Portal (https://dcc.icgc.org/pcawg) upon request.

## Code availability

The codes were available through the github at https://github.com/dalcsy/NEER-cancer-exceptional-responders-PRS-analysis.

## References

[1] Wheeler DA (2021). Molecular features of cancers exhibiting exceptional responses to treatment. Cancer Cell.

[2] Bilusic M (2021). Molecular profiling of exceptional responders to cancer therapy. Oncologist.

[3] Conley BA (2021). The exceptional responders initiative: feasibility of a National Cancer Institute Pilot Study. J. Natl Cancer Inst..

[4] Iyer G (2012). Genome sequencing identifies a basis for everolimus sensitivity. Science.

[5] Cooper AJ (2020). Identification of a RAS-activating TMEM87A-RASGRF1 fusion in an exceptional responder to sunitinib with non-small cell lung cancer. Clin. Cancer Res..

[6] Pagadala M (2023). Germline modifiers of the tumor immune microenvironment implicate drivers of cancer risk and immunotherapy response. Nat. Commun..

[7] Choi J (2022). Polygenic risk scores associated with tumor immune infiltration in common cancers. Cancers.

[8] Khan Z (2021). Genetic variation associated with thyroid autoimmunity shapes the systemic immune response to PD-1 checkpoint blockade. Nat. Commun..

[9] Mansour Aly D (2021). Genome-wide association analyses highlight etiological differences underlying newly defined subtypes of diabetes. Nat. Genet..

[10] Privé F (2022). Portability of 245 polygenic scores when derived from the UK Biobank and applied to 9 ancestry groups from the same cohort. Am. J. Hum. Genet..

[11] Barnes CLK (2021). Contribution of common risk variants to multiple sclerosis in Orkney and Shetland. Eur. J. Hum. Genet..

[12] Chun S (2020). Non-parametric polygenic risk prediction via partitioned GWAS Summary Statistics. Am. J. Hum. Genet..

[13] Luo J (2021). Immunotherapy-mediated thyroid dysfunction: genetic risk and impact on outcomes with PD-1 blockade in non-small cell lung cancer. Clin. Cancer Res..

[14] Sayaman RW (2021). Germline genetic contribution to the immune landscape of cancer. Immunity.

[15] ICGC/TCGA Pan-Cancer Analysis of Whole Genomes Consortium. Pan-cancer analysis of whole genomes. *Nature* **578**, 82–93 (2020). 10.1038/s41586-020-1969-6 PMC7025898 32025007

[16] Sohn E (2021). Why autoimmunity is most common in women. Nature.

[17] Hoos A (2016). Development of immuno-oncology drugs - from CTLA4 to PD1 to the next generations. Nat. Rev. Drug Discov..

[18] Zhou Y (2021). Knockdown of CENPW inhibits hepatocellular carcinoma progression by inactivating E2F signaling. Technol. Cancer Res. Treat..

[19] Su H, Fan Y, Wang Z, Jiang L (2022). A comprehensive investigation on pan-cancer impacts of constitutive centromere associated network gene family by integrating multi-omics data: a CONSORT-compliant article. Medicine.

[20] Cai M (2021). A unified framework for cross-population trait prediction by leveraging the genetic correlation of polygenic traits. Am. J. Hum. Genet..

[21] Ruan Y (2022). Improving polygenic prediction in ancestrally diverse populations. Nat. Genet..

[22] Wang K (2010). Comparative genetic analysis of inflammatory bowel disease and type 1 diabetes implicates multiple loci with opposite effects. Hum. Mol. Genet..

[23] Cubas, R. et al. Autoimmunity linked protein phosphatase PTPN22 as a target for cancer immunotherapy. *J. Immunother. Cancer* **8** (2020). 10.1136/jitc-2020-001439 PMC7604869 33127657

[24] Wang H (2022). E3 ubiquitin ligase MAGI3 degrades c-Myc and acts as a predictor for chemotherapy response in colorectal cancer. Mol. Cancer.

[25] Vivarelli, S., Falzone, L., Candido, S., Bonavida, B. & Libra, M. YY1 silencing induces 5-fluorouracil-resistance and BCL2L15 downregulation in colorectal cancer cells: diagnostic and prognostic relevance. *Int. J. Mol. Sci*. **22** (2021). 10.3390/ijms22168481 PMC8395225 34445183

[26] Khan Z (2020). Polygenic risk for skin autoimmunity impacts immune checkpoint blockade in bladder cancer. Proc. Natl. Acad. Sci. USA..

[27] American Cancer Society. *Cancer Facts & Figures* (American Cancer Society, 2020). 10.6004/jadpro.2020.11.2.1 PMC7848816 33532112

[28] *National Cancer Institute* (National Cancer Institute, Office of Cancer Communications, 2020).

[29] Lee BT (2022). The UCSC Genome Browser database: 2022 update. Nucleic Acids Res..

[30] De Marino A (2022). A comparative analysis of current phasing and imputation software. PLoS ONE.

[31] Das S (2016). Next-generation genotype imputation service and methods. Nat. Genet..

[32] Hung RJ (2021). Assessing lung cancer absolute risk trajectory based on a polygenic risk model. Cancer Res..

[33] Lam M (2019). Comparative genetic architectures of schizophrenia in East Asian and European populations. Nat. Genet..

[34] Martin AR (2019). Clinical use of current polygenic risk scores may exacerbate health disparities. Nat. Genet..

[35] Duncan L (2019). Analysis of polygenic risk score usage and performance in diverse human populations. Nat. Commun..

[36] Shapiro SS, Wilk MB, Chen HJ (1968). A comparative study of various tests for normality. J. Am. Stat. Assoc..

