# Supplementary material for: Polygenic risk scores for autoimmune related diseases are significantly different in cancer exceptional responders
Source: NPJ Precis Oncol. 2024 May 25;8:120. doi: 10.1038/s41698-024-00613-x (PMC11127926; doi:10.1038/s41698-024-00613-x)
Supplement: Supplementary file 1 — Supplementary Information [file 41698_2024_613_MOESM1_ESM.pdf]

Supplementary Figures

Supplementary Figure 1. Histograms of 7 autoimmune related disease PRSs in NEER and PCAWG.

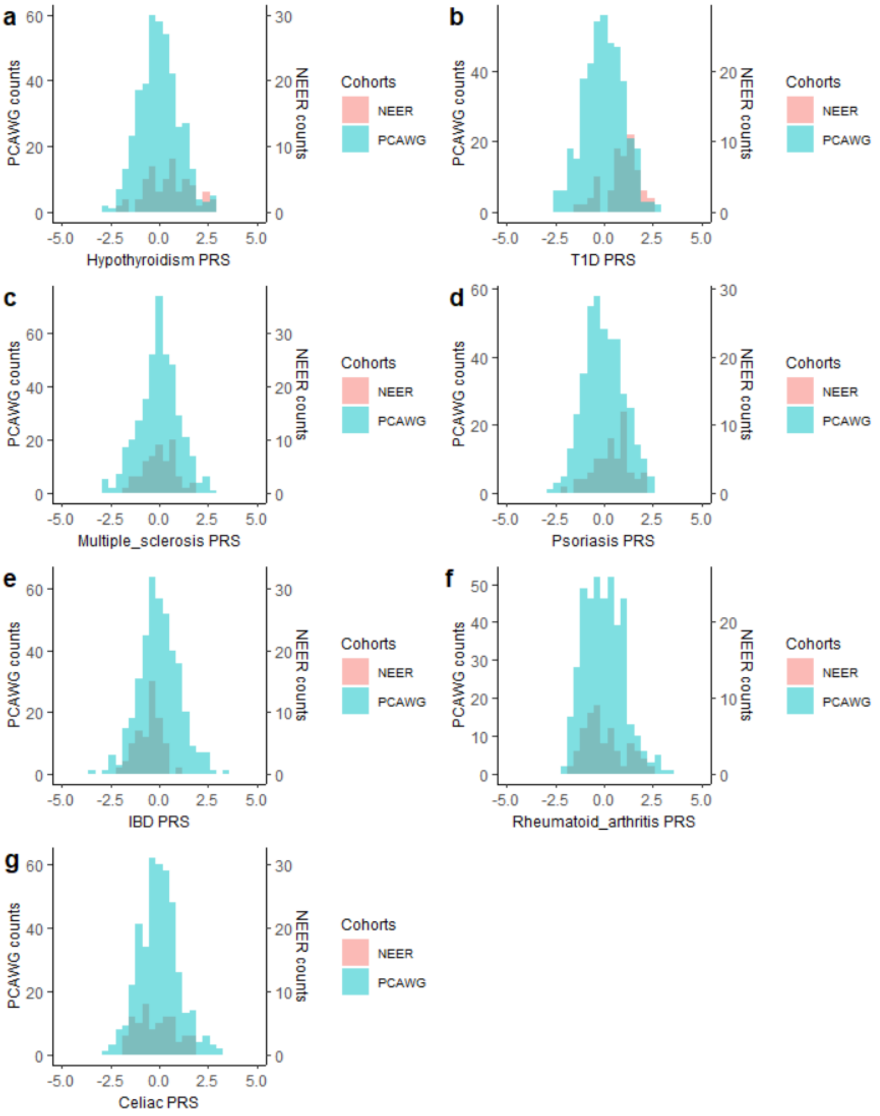

The histograms show the distribution of PRSs without being smoothed. The main y axis (left) shows counts of PCAWG typical patients with the given binned PRS, while the second y axis (right) shows the counts of NEER ERs.

Supplementary Figure 2. Survival curves of PCAWG patients stratified by high vs low PRSs

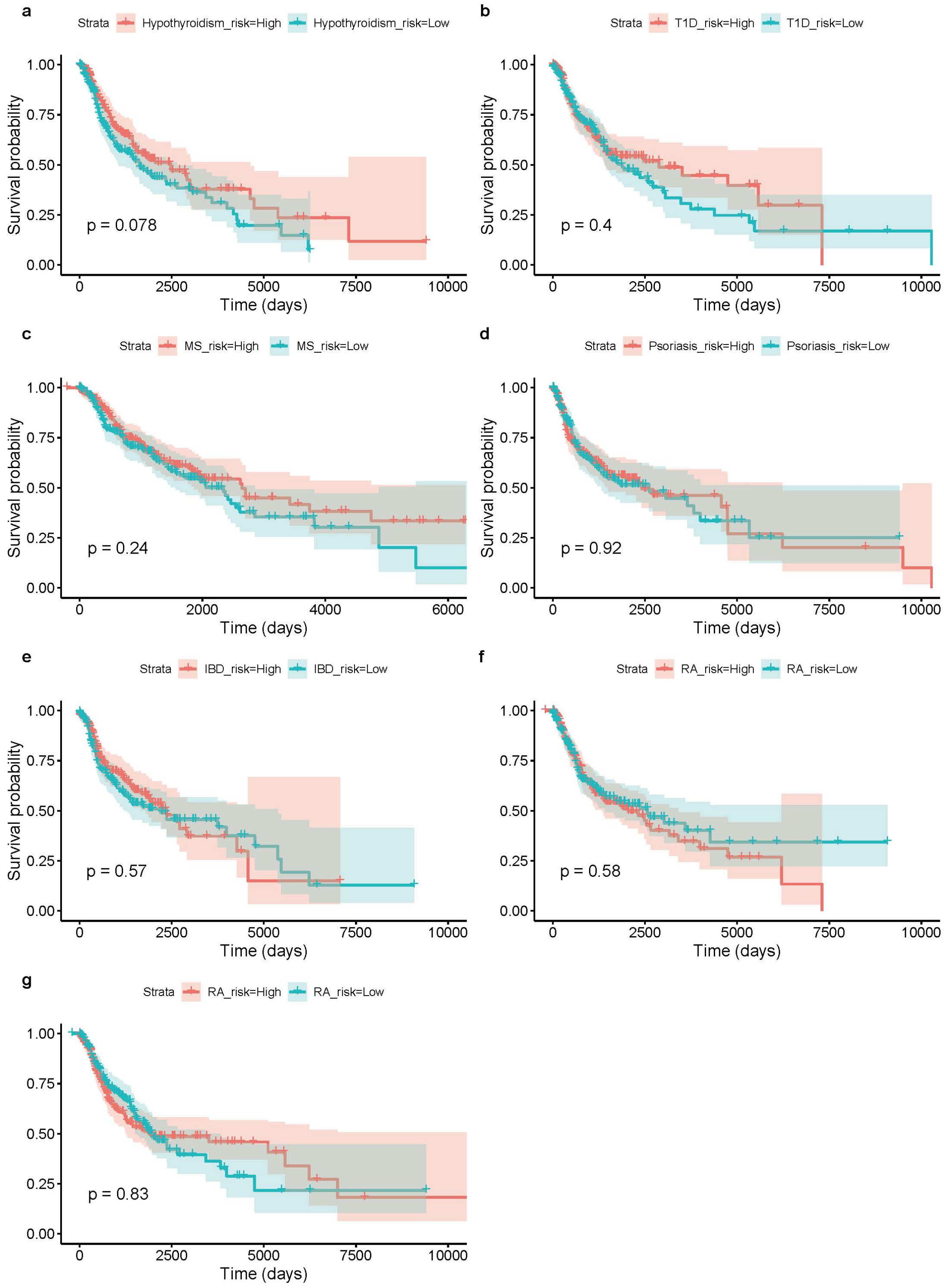

Survival curves in PCAWG typical cancer patients, stratified by PRSs in 7 autoimmune diseases. The red curve refers to those patients with high PRSs and high risk of diseases, while the blue curve refers to those with low risks. The number of individuals followed up for each time interval is shown in the table.

Supplementary Figure 3. PRS distributions stratified by different types of therapy

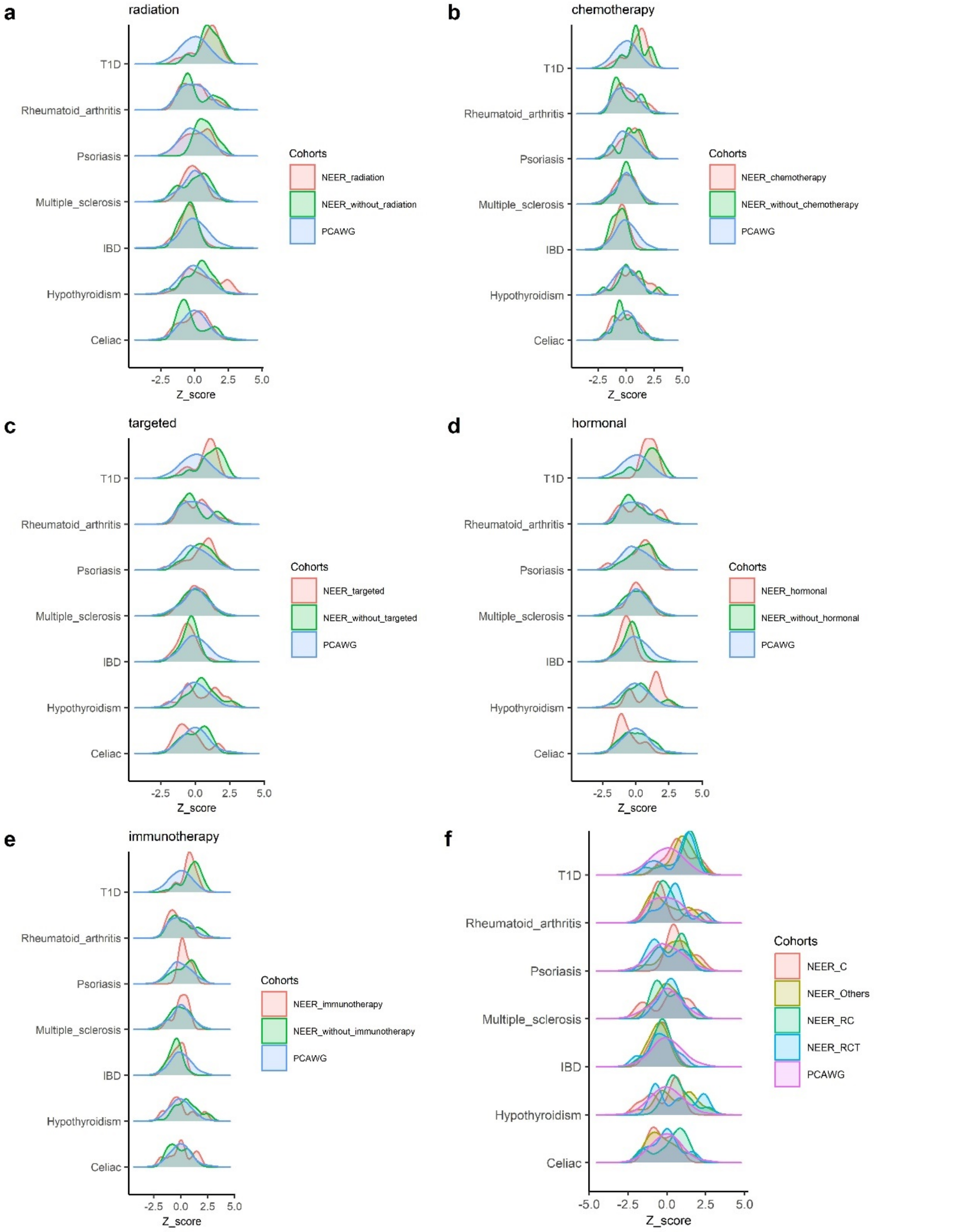

A-E) PRS distributions of seven autoimmune diseases, with NEER ERs stratified by whether received a specific type of therapy. F) PRS distributions of seven autoimmune diseases, with NEER ERs stratified by regimens (combination of therapies). NEER_C (n=7), NEER_RC (n=10) and NEER_RCT (n=7) refer to chemotherapy only, a combination of radiation therapy and chemotherapy, and a combination of radiation therapy, chemotherapy and targeted therapy respectively among NEER ERs. NEER_Others refers to the group of ERs with other combinations of therapies. Each specific combination has a sample size less than 7.

Supplementary Figure 4. 2-D PCA plot of all NEER and PCAWG patients.

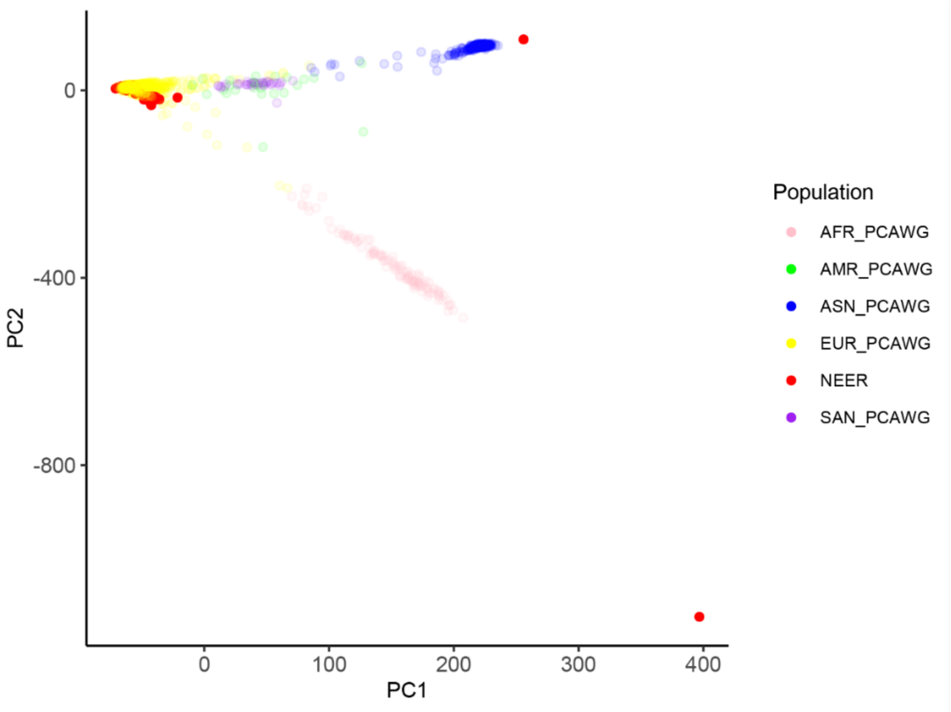

PCA was conducted among all NEER and PCAWG patients and PCAWG patients were colored by previously reported ancestry populations.

Supplementary Methods

Study inclusion reasons for those were decided individually

Participant 1: Stopped conventional treatments after 3 years and began an anti-angiogenic diet, resulting in cancer resolving and being NED for years.

Participant 6: Was a complete responder to an IDH targeted drug and stem cell transplants.

Participant 13: Had widespread cancer but remained cancer-free after maintenance therapy for over a year.

Participant 20: Responded well to Keytruda for a rare type of melanoma not usually affected by that drug.

Participant 28: Showed no evidence of recurrence for years after chemotherapy (in a failed clinical trial) for metastatic breast cancer.

Participant 33: Greatly improved after using cannabis oil despite dire prognosis for widely metastatic breast cancer.

Participant 51: Avoided systemic treatment and had no new metastases for over 6 years after kidney cancer surgery, unique case.

Supplementary Tables

Supplementary Table 1

	**NEER**
N	51
Treatment Class, n(%)	
Surgery	40 (78.4%)
Chemotherapy	38 (74.5%)
Radiation	30 (58.8%)
Targeted	23 (45.1%)
Hormonal	11 (21.6%)
Immunotherapy	7 (13.7%)

Supplementary Table 2

Phenotype	Number of SNPs	Cite
Hypothyroidism	1099649	Khan, Zia, et al. "Genetic variation associated with thyroid autoimmunity shapes the systemic immune response to PD-1 checkpoint blockade." *Nature communications* 12.1 (2021): 1-12.
T1D	66	Mansour Aly, Dina, et al. "Genome-wide association analyses highlight etiological differences underlying newly defined subtypes of diabetes." *Nature genetics* 53.11 (2021): 1534-1542.
Multiple sclerosis	127	Barnes, Catriona LK, et al. "Contribution of common risk variants to multiple sclerosis in Orkney and Shetland." *European Journal of Human Genetics* 29.11 (2021): 1701-1709.
Psoriasis	71744	Privé, Florian, et al. "Portability of 245 polygenic scores when derived from the UK Biobank and applied to 9 ancestry groups from the same cohort." *The American Journal of Human Genetics* 109.1 (2022): 12-23.
Rheumatoid arthritis	256	Privé, Florian, et al. "Portability of 245 polygenic scores when derived from the UK Biobank and applied to 9 ancestry groups from the same cohort." *The American Journal of Human Genetics* 109.1 (2022): 12-23.
IBD	5784395	Chun, Sung, et al. "Non-parametric polygenic risk prediction via partitioned GWAS summary statistics." *The American Journal of Human Genetics* 107.1 (2020): 46-59.
Celiac	58231	Privé, Florian, et al. "Portability of 245 polygenic scores when derived from the UK Biobank and applied to 9 ancestry groups from the same cohort." *The American Journal of Human Genetics* 109.1 (2022): 12-23.

Supplementary Table 3

id	gender	organ_system	age	donor_vital_status	cancer_stage	autoimmune_disease	family_autoimmune	surgery	radiation	chemotherapy	targeted	hormonal	immunotherapy	years_survived
1	F	BREAST	55	alive	IV	Yes	No	Yes	Yes	Yes	Yes	Yes	No	16.41
2	M	BLOOD, BONE MARROW, & HEMATOPOIETIC SYS	55	alive	Binet C	No	NA	Yes	No	No	No	No	No	29.95
3	M	PROSTATE GLAND	62	alive	IV	No	No	Yes	Yes	No	No	Yes	No	10.44
4	M	URINARY BLADDER	57	alive	IV	No	No	Yes	Yes	Yes	No	No	No	7.84
5	M	PANCREAS	75	alive	IV	No	No	No	Yes	Yes	Yes	No	No	6.31
6	M	BLOOD, BONE MARROW, & HEMATOPOIETIC SYS	65	alive	NA	No	No	No	Yes	Yes	Yes	No	No	9.11
7	M	PANCREAS	69	alive	IV	No	No	Yes	No	Yes	No	No	No	8.71
8	M	SKIN	65	alive	IV	Yes	Yes	Yes	Yes	No	No	No	No	12.44
9	F	OVARY	82	alive	IV	No	Yes	Yes	No	Yes	No	No	No	30.77
10	F	BREAST	66	alive	IV	Yes	No	Yes	Yes	Yes	Yes	Yes	Yes	25.68
11	F	LUNG & BRONCHUS	62	alive	IV	No	Yes	Yes	Yes	Yes	Yes	No	No	7.10

12	F	OVARY	66	alive	IV	No	No	Yes	No	Yes	No	No	No	19.24
13	F	OVARY	66	alive	IV	No	NA	Yes	No	Yes	Yes	Yes	No	11.21
14	M	KIDNEY	63	deceased	IV	Yes	No	No	Yes	No	Yes	No	Yes	15.71
15	F	LIVER OR GALLBLADDER	50	alive	IV	No	No	Yes	No	Yes	No	No	Yes	11.19
16	F	UTERUS	64	alive	IV	Yes	No	Yes	Yes	Yes	No	No	No	10.32
17	F	BREAST	61	alive	IV	No	No	No	Yes	Yes	Yes	No	No	10.55
18	F	PANCREAS	43	alive	IV	Yes	Yes	Yes	No	Yes	No	No	No	5.86
19	F	BREAST	70	alive	IV	No	No	Yes	Yes	Yes	Yes	Yes	No	12.98
20	F	SKIN	39	alive	IV	Yes	No	Yes	Yes	No	No	No	Yes	3.15
21	M	BONE & SOFT TISSUE	66	alive	III	No	No	Yes	Yes	No	No	No	No	28.31
22	M	BLOOD, BONE MARROW, & HEMATOPOIETIC SYS	66	alive	NA	No	No	No	No	No	Yes	No	No	7.06
23	F	ESOPHAGUS OR STOMACH	76	alive	IV	No	No	No	No	Yes	No	No	No	9.68
24	F	LUNG & BRONCHUS	72	alive	IV	NA	NA	NA	NA	Yes	Yes	NA	NA	8.92
25	F	LUNG & BRONCHUS	67	alive	IV	No	No	Yes	Yes	No	Yes	No	No	15.85
26	F	BREAST	57	deceased	IV	NA	NA	NA	NA	Yes	NA	NA	NA	8.29

27	F	BREAST	70	alive	IV	No	No	Yes	Yes	Yes	Yes	No	NA	21.42
28	F	BREAST	66	alive	IV	No	No	Yes	Yes	Yes	No	No	No	24.62
29	M	BRAIN, & CRANIAL NERVES, & SPINAL CORD, (EXCL. VENTRICLE, CEREBELLUM)	67	alive	NA	No	No	Yes	Yes	Yes	No	No	No	21.86
30	F	BREAST	73	alive	IV	No	No	Yes	Yes	Yes	No	Yes	No	31.18
31	M	PANCREAS	62	alive	IV	No	No	Yes	Yes	Yes	No	No	No	7.27
32	M	BRAIN, & CRANIAL NERVES, & SPINAL CORD, (EXCL. VENTRICLE, CEREBELLUM)	76	decease d	NA	No	No	Yes	Yes	Yes	Yes	No	Yes	8.53
33	F	BREAST	60	alive	IV	No	No	Yes	Yes	Yes	Yes	No	No	14.42
34	M	PANCREAS	69	alive	IV	No	No	Yes	No	Yes	Yes	No	No	4.31
35	F	LUNG & BRONCHUS	69	alive	IV	Yes	No	Yes	Yes	Yes	Yes	No	No	12.77
36	F	KIDNEY	70	alive	IV	No	Yes	Yes	No	No	Yes	No	Yes	15.96
37	F	UTERUS	81	alive	IV	No	No	Yes	No	Yes	No	No	No	16.11

38	F	BREAST	76	alive	IV	Yes	Yes	Yes	No	Yes	No	Yes	No	30.12
39	M	LARGE INTESTINE	70	alive	IV	No	No	Yes	No	Yes	Yes	No	No	16.94
40	F	OVARY	75	alive	IV	No	No	Yes	No	Yes	No	Yes	No	16.78
41	M	LARGE INTESTINE	79	alive	IV	NA	NA	Yes	Yes	Yes	No	No	No	12.88
42	M	PANCREAS	87	alive	III	No	Yes	Yes	Yes	Yes	No	No	No	19.14
43	F	BREAST	67	alive	IV	No	No	Yes	Yes	Yes	Yes	Yes	No	30.65
44	F	BLOOD, BONE MARROW, & HEMATOPOIE TIC SYS	67	alive	NA	Yes	No	No	No	No	Yes	No	No	10.01
45	M	THYROID GLAND	54	alive	IV	No	No	Yes	Yes	Yes	No	No	Yes	11.17
46	F	UTERUS	66	alive	IV	No	No	Yes	Yes	Yes	No	No	No	10.67
47	F	LUNG & BRONCHUS	66	alive	IV	No	Yes	Yes	Yes	Yes	No	No	No	11.44
48	M	PANCREAS	71	alive	IV	No	No	Yes	Yes	Yes	No	No	No	14.10
49	F	BREAST	65	decease d	IV	NA	NA	NA	NA	NA	Yes	Yes	NA	19.42
50	F	BREAST	73	alive	IV	NA	NA	NA	NA	NA	Yes	Yes	NA	15.71
51	M	KIDNEY	55	alive	IV	No	No	Yes	No	No	No	No	No	8.16

Supplementary Table 4

snp_id	eQTL	Nearest gene	functional_consequence	chr	position	effect_weight	n_alt_alleles_pcawg	percentage_alt_alleles_pcawg	n_alt_alleles_neer	percentage_alt_alleles_neer	odds_ratio (PCAWG/NEER)	p	p.adj
rs10840442	-	IGF2, INS-IGF2	intron_variant, upstream_transcript_variant, 2KB_upstream_variant	11	2171922	0.153	142	0.171	16	0.157	1.112	0.781	0.998
rs11066320	ALDH2	PTPN11, LOC124903023	intron_variant	12	112906415	0.209	463	0.559	51	0.500	1.268	0.291	0.844
rs11203203	UBASH3A	UBASH3A	5_prime_UTR_variant,intron_variant	21	43836186	0.150	320	0.386	38	0.373	1.061	0.830	1.000
rs113010081	CCR3	None		3	46457412	-0.164	90	0.109	8	0.078	1.432	0.398	0.861
rs117693013	TRPM5	None		11	2198479	-0.421	44	0.053	3	0.029	1.851	0.469	0.861
rs12128454	PTPN22	None		1	113910209	0.274	87	0.105	11	0.108	0.971	0.865	1.000
rs12150079	GSDMB	ZPBP2	intron_variant	17	38025417	0.114	263	0.318	38	0.373	0.784	0.264	0.844

rs12284746	C11orf21	None		11	2277229	0.182	65	0.079	15	0.147	0.495	0.037	0.606
rs12416116	RNLS	RNLS, LOC101929727	intron_variant, genic_upstream_transcript_variant,genic_downstream_transcript_variant	10	90035654	-0.165	242	0.292	36	0.353	0.757	0.209	0.844
rs1456988	-	None		14	98488007	0.111	608	0.734	70	0.686	1.263	0.345	0.861
rs1503836	DCLRE1B	None		1	114541498	0.145	346	0.418	44	0.431	0.946	0.832	1.000
rs151233	TUFM	APOBR	synonymous_variant,coding_sequence_variant	16	28506428	0.171	108	0.130	22	0.216	0.546	0.023	0.514
rs1539438	AP4B1	BCL2L15,AP4B1-AS1	intron_variant, upstream_transcript_variant, 2KB_upstream_variant	1	114430356	-0.157	548	0.662	65	0.637	1.114	0.658	0.966
rs1574285	-	GLIS3	intron_variant, genic_upstream_transcript_variant	9	4283137	0.115	466	0.563	55	0.539	1.100	0.673	0.966

rs1701704	RAB5B	IKZF4, LOC105369781	intron_variant, genic_upstream_transcript_variant	12	56412487	0.223	264	0.319	28	0.275	1.237	0.429	0.861
rs17630235	ALDH2	TRAFD1	500B_downstream_variant,downstream_transcript_variant	12	112591686	0.222	353	0.426	51	0.500	0.743	0.169	0.844
rs1893217	-	PTPN2	intron_variant	18	12809340	0.192	116	0.140	14	0.137	1.024	1.000	1.000
rs193779	RMI2	SOCS1, LOC105371082	intron_variant, 2KB_upstream_variant,genic_upstream_transcript_variant,upstream_transcript_variant	16	11350965	0.129	211	0.255	25	0.245	1.053	0.904	1.000
rs2045258	CENPW	CENPW	intron_variant, genic_downstream_transcript_variant	6	126674354	0.117	397	0.479	58	0.569	0.699	0.094	0.844
rs2111485	-	LOC105373724	genic_downstream_transcript_variant,intron_variant	2	163110536	-0.165	488	0.589	62	0.608	0.926	0.750	0.998

rs229533	C1QTNF6	C1QTNF6	genic_upstream_transcript_variant,intron_variant	22	37587111	0.107	387	0.467	52	0.510	0.844	0.462	0.861
rs2304256	TYK2	TYK2	missense_variant,coding_sequence_variant,genic_upstream_transcript_variant,intron_variant,upstream_transcript_variant	19	10475652	-0.139	242	0.292	24	0.235	1.342	0.247	0.844
rs3087243	CTLA4	CTLA4	500B_downstream_variant,downstream_transcript_variant	2	204738919	-0.178	395	0.477	41	0.402	1.357	0.172	0.844
rs3184504	SH2B3	SH2B3	synonymous_variant,missense_variant,coding_sequence_variant,5_prime_UTR_variant	12	111884608	-0.266	437	0.528	46	0.451	1.360	0.172	0.844
rs34593439	CTSH	CTSH	intron_variant	15	79234957	-0.246	84	0.101	8	0.078	1.326	0.598	0.953

rs34835	SPNS1	CLN3	intron_variant	16	28499291	-0.121	349	0.421	38	0.373	1.227	0.395	0.861
rs3842727	C11orf21	TH	downstream_transcript_variant,500B_downstream_variant	11	2184848	-0.687	563	0.680	76	0.745	0.727	0.213	0.844
rs3842763	-	INS-IGF2	intron_variant	11	2179204	0.149	232	0.280	29	0.284	0.980	0.908	1.000
rs402072	FKRP	PRKD2	genic_upstream_transcript_variant,upstream_transcript_variant,5_prime_UTR_variant, intron_variant	19	47219122	-0.142	140	0.169	17	0.167	1.017	1.000	1.000
rs4378452	GPN3	CUX2	intron_variant, genic_upstream_transcript_variant	12	111504033	0.144	281	0.339	43	0.422	0.705	0.123	0.844
rs4766897	SH2B3	ACAD10	intron_variant	12	112179471	0.189	569	0.687	66	0.647	1.198	0.431	0.861
rs4767000	OAS1	RPH3A	intron_variant, genic_upstream_transcript_variant	12	113168368	0.116	453	0.547	45	0.441	1.529	0.046	0.606

rs4820830	MTMR3	HORMAD2	intron_variant	22	30531091	0.135	521	0.629	59	0.578	1.237	0.331	0.861
rs4839318	-	None		1	113918302	-0.113	301	0.364	31	0.304	1.308	0.274	0.844
rs4849135	-	ACOXL	intron_variant	2	111615079	-0.115	599	0.723	74	0.725	0.990	1.000	1.000
rs516246	FUT2	FUT2, LOC105447645	non_coding_transcript_variant,intron_variant	19	49206172	-0.143	374	0.452	59	0.578	0.601	0.020	0.514
rs56994090	-	MEG3	intron_variant	14	101306447	-0.129	347	0.419	47	0.461	0.844	0.458	0.861
rs5752973	MTMR3	None		22	30264274	0.123	331	0.400	47	0.461	0.780	0.242	0.844
rs6043409	SIRPG	SIRPG, SIRPG-AS1	3_prime_UTR_variant,missense_variant,intron_variant,coding_sequence_variant	20	1616206	-0.126	544	0.657	74	0.725	0.725	0.183	0.844
rs61819372	-	MAGI3	intron_variant	1	114072110	0.428	7	0.008	1	0.010	0.861	0.607	0.953
rs61839660	IL2RA	IL2RA	intron_variant	10	6094697	-0.472	66	0.080	11	0.108	0.717	0.340	0.861
rs62447205	IKZF1	IKZF1	intron_variant, genic_downstr	7	50465830	-0.117	212	0.256	25	0.245	1.060	0.904	1.000

			eam_transcript_variant										
rs6578997	C11orf21	None		11	2220414	-0.320	75	0.091	7	0.069	1.351	0.580	0.953
rs6669008	PHTF1	MAGI3	intron_variant	1	114166561	-0.161	351	0.424	49	0.480	0.796	0.290	0.844
rs6679677	PTPN22	PHTF1	2KB_upstream_variant,upstream_transcript_variant	1	114303808	0.636	82	0.099	12	0.118	0.825	0.600	0.953
rs6691977	DDX59	CAMSAP2	intron_variant	1	200814959	0.126	173	0.209	21	0.206	1.019	1.000	1.000
rs6827756	KIAA1109	KIAA1109	intron_variant	4	123184411	0.131	507	0.612	63	0.618	0.978	1.000	1.000
rs6836189	-	IL21, IL21-AS1	intron_variant	4	123541341	0.127	480	0.580	59	0.578	1.005	1.000	1.000
rs7073236	IL2RA	None		10	6106552	0.190	347	0.419	40	0.392	1.118	0.671	0.966
rs7239671	DOK6	CD226	genic_downstream_transcript_variant,3_prime_UTR_variant	18	67523260	0.121	397	0.479	43	0.422	1.264	0.294	0.844
rs72687906	MAGI3	None		1	113922269	0.480	25	0.030	2	0.020	1.556	0.759	0.998

rs72687939	PTPN22	MAGI3	intron_variant	1	114011306	0.180	123	0.149	15	0.147	1.012	1.000	1.000
rs72727394	RASGRP1	RASGRP1	intron_variant	15	38847022	0.138	176	0.213	18	0.176	1.259	0.441	0.861
rs72853903	TH	None		11	2198665	-0.278	233	0.281	26	0.255	1.144	0.640	0.966
rs72853956	TSPAN32	None		11	2222271	-0.421	32	0.039	3	0.029	1.326	1.000	1.000
rs72928038	BACH2	BACH2	intron_variant	6	90976768	0.180	151	0.182	20	0.196	0.915	0.786	0.998
rs7396243	TH	None		11	2205892	-0.295	492	0.594	65	0.637	0.834	0.454	0.861
rs741172	DEXI	CLEC16A	intron_variant, genic_downstream_transcript_variant	16	11200798	-0.194	276	0.333	28	0.275	1.321	0.264	0.844
rs7480143	-	None		11	2225023	0.171	109	0.132	24	0.235	0.493	0.007	0.458
rs77347828	-	LOC101928080	intron_variant	10	6168290	0.159	106	0.128	14	0.137	0.923	0.756	0.998
rs7893467	-	IL2RA	intron_variant	10	6079035	0.196	763	0.921	90	0.882	1.564	0.182	0.844
rs7925375	C11orf21	TH	intron_variant	11	2191155	0.178	631	0.762	74	0.725	1.212	0.462	0.861
rs8056814	CFDP1	CTRB1	2KB_upstream_variant,upstre	16	75252327	0.278	76	0.092	11	0.108	0.836	0.589	0.953

			am_transcript_variant										
rs853981	CENPW	None		6	127045460	-0.107	404	0.488	42	0.412	1.361	0.172	0.844
rs9585056	TM9SF2	None		13	100081766	0.116	613	0.740	74	0.725	1.079	0.722	0.998
rs977986	CFDP1	CHST6	non_coding_transcript_variant,3_prime_UTR_variant	16	75506696	0.171	701	0.847	84	0.824	1.183	0.563	0.953

Supplementary Table 5

snp_id	gene	odds_ratio	p_value	p_adjust	Function to T1D
rs6669008	MAGI3	0.319	0.0013	0.074	protective
rs1539438	BCL2L15	4.64	0.0058	0.095	susceptible
rs3842727	TH	5.202	0.0026	0.074	susceptible
rs3184504	SH2B3	0.179	0.0033	0.074	protective
